# Supplementary figures and images for: Prevalence and risk factors of COVID-19-related generalized anxiety disorder among the general public in China: a cross-sectional study
Source: PeerJ. 2023 Jan 18;11:e14720. doi: 10.7717/peerj.14720 (PMC9864122; doi:10.7717/peerj.14720)

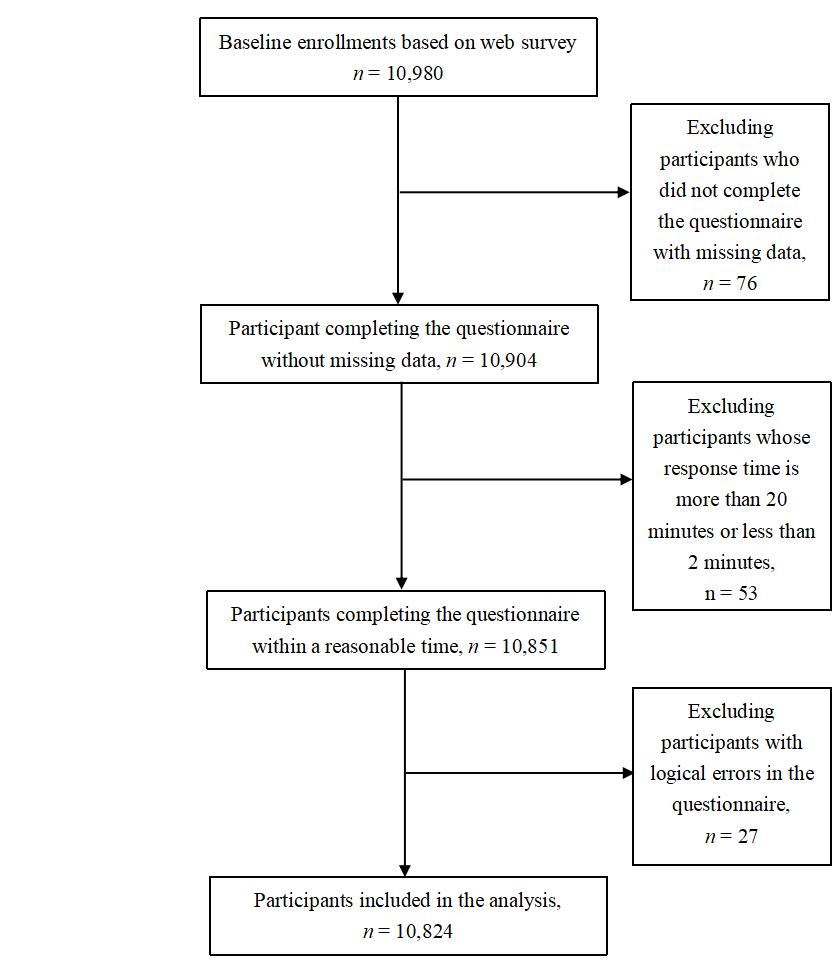

Supplement: Supplemental Information 1 [file peerj-11-14720-s001.jpg]

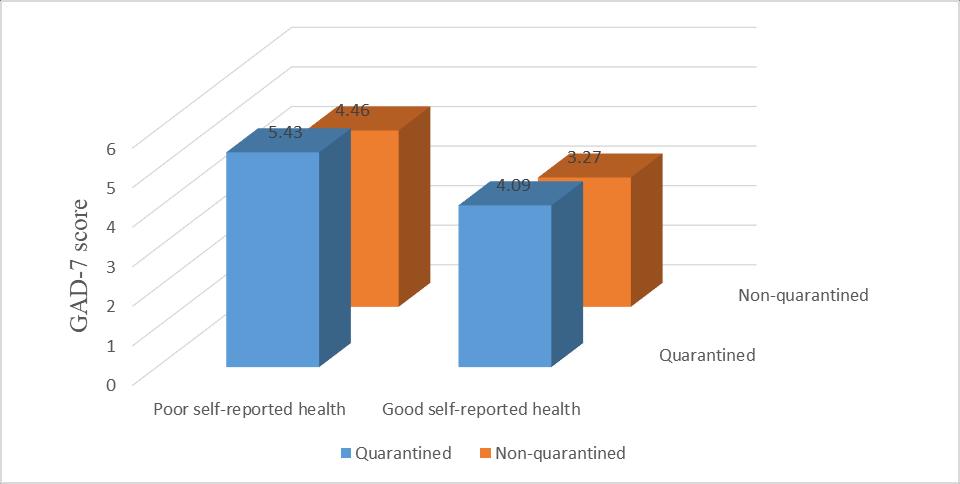

Supplement: Supplemental Information 2 [file peerj-11-14720-s002.jpg]

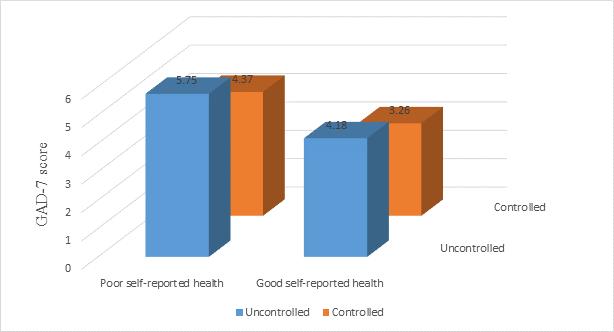

Supplement: Supplemental Information 3 [file peerj-11-14720-s003.jpg]
