# Supplementary material for: Prevalence and risk factors of COVID-19-related generalized anxiety disorder among the general public in China: a cross-sectional study
Source: PeerJ. 2023 Jan 18;11:e14720. doi: 10.7717/peerj.14720 (PMC9864122; doi:10.7717/peerj.14720)
Supplement: Supplemental Information 4 [file peerj-11-14720-s004.docx]

**Supplemental Table 1 The number of people surveyed in each area**

| **Eastern** | | **Central** | | **Western** | |
| --- | --- | --- | --- | --- | --- |
| **Province** | **Number** | **Province** | **Number** | **Province** | **Number** |
| Beijing | 117 | Shanxi | 115 | Gansu | 604 |
| Tianjin | 33 | Henan | 1256 | Yunnan | 313 |
| Hebei | 333 | Hunan | 910 | Guizhou | 210 |
| Shanghai | 188 | Hubei | 635 | Qinghai | 205 |
| Jiangsu | 387 | Anhui | 408 | Ningxia | 163 |
| Zhejiang | 509 | Jiangxi | 355 | Xinjiang | 121 |
| Fujian | 257 |  |  | Tibet | 1 |
| Shandong | 493 |  |  | Chongqing | 14 |
| Guangdong | 1220 |  |  | Guangxi | 318 |
| Hainan | 15 |  |  | Sichuan | 295 |
| Liaoning | 136 |  |  | Shaanxi | 189 |
| Jilin | 262 |  |  | Inner Mongolia | 312 |
| Heilongjiang | 441 |  |  |  |  |
| Taiwan | 6 |  |  |  |  |
| Hong Kong | 2 |  |  |  |  |
| Macao | 1 |  |  |  |  |
