# Supplementary material for: Prevalence and risk factors of COVID-19-related generalized anxiety disorder among the general public in China: a cross-sectional study
Source: PeerJ. 2023 Jan 18;11:e14720. doi: 10.7717/peerj.14720 (PMC9864122; doi:10.7717/peerj.14720)
Supplement: Supplemental Information 5 — Notes: GAD-7, Generalized Anxiety Disorder-Scale-7; COVID-19: Coronavirus Disease 2019; p < 0.05, statistically significant. [file peerj-11-14720-s005.docx]

**Supplemental Table 2 Associations between anxiety symptoms (GAD-7 score) with demographic characteristics, health status and COVID-19-related variables in eastern, central and western China**

| **Variable** | **Categories** | **Eastern China** | | | | **Central China** | | | | **Western China** | | | |
| --- | --- | --- | --- | --- | --- | --- | --- | --- | --- | --- | --- | --- | --- |
|  |  | **Anxiety symptoms (GAD-7 score)** | | **χ2** | **p** | **Anxiety symptoms (GAD-7 score)** | | **χ2** | **p** | **Anxiety symptoms**  **(GAD-7 score)** | | **χ2** | **p** |
|  |  | **Yes ( ≥ 5)** | **No ( < 5)** |  |  | **Yes ( ≥ 5)** | **No ( < 5)** |  |  | **Yes ( ≥ 5)** | **No ( < 5)** |  |  |
| Gender | Female | 865(36.7%) | 1,491(63.3%) | 0.386 | 0.535 | 788(40.8%) | 1,145(59.2%) | 14.581 | < .001 | 651(42.3%) | 887(57.7%) | 10.022 | 0 .002 |
|  | Male | 732(35.8%) | 1,312(64.2%) |  |  | 605(34.7%) | 1,141(65.3%) |  |  | 439(36.4%) | 768(63.6%) |  |  |
| Age | 39 or below | 1,171(38.7%) | 1,857(61.3%) | 23.728 | < .001 | 1,019(39.3%) | 1,577(60.7%) | 7.233 | 0.007 | 834(40.8%) | 1208(59.2%) | 4.281 | 0.039 |
|  | 40 or above | 426(31.0%) | 946(69.0%) |  |  | 374(34.5%) | 709(65.5%) |  |  | 256(36.4%) | 447(63.6%) |  |  |
| Marital status | Unmarried | 432(38.6%) | 688(61.4%) | 3.366 | 0.067 | 322(37.4%) | 539(62.6%) | 0.103 | 0.748 | 316(42.5%) | 427(57.5%) | 3.388 | 0.066 |
|  | Married | 1,165(35.5%) | 2,115(64.5%) |  |  | 1,071(38.0%) | 1,747(62.0%) |  |  | 774(38.7%) | 1,228(61.3%) |  |  |
| Family register | Rural | 333(41.4%) | 471(58.6%) | 11.164 | < .001 | 441(40.1%) | 659(59.9%) | 3.309 | 0.069 | 355(43.3%) | 465(56.7%) | 6.274 | 0.012 |
|  | Urban | 1,264(35.2%) | 2,332(64.8%) |  |  | 952(36.9%) | 1627(63.1%) |  |  | 735(38.2%) | 1,190(61.8%) |  |  |
| Educational level | Secondary or below | 390(35.3%) | 714(64.7%) | 0.599 | 0.439 | 614(37.4%) | 1,027(62.6%) | 0.252 | 0.616 | 421(41.1%) | 604(58.9%) | 1.272 | 0.259 |
|  | Tertiary or above | 1,207(36.6%) | 2,089(63.4%) |  |  | 779(38.2%) | 1,259(61.8%) |  |  | 669(38.9%) | 1,051(61.1%) |  |  |
| Occupational background | Medical background | 599(35.1%) | 1,108(64.9%) | 1.75 | 0.186 | 424(37.6%) | 703(62.4%) | 0.04 | 0.841 | 348(39.1%) | 543(60.9%) | 0.234 | 0.629 |
|  | Non-medical background | 998(37.1%) | 1,695(62.9%) |  |  | 969(38.0%) | 1583(62.0%) |  |  | 742(40.0%) | 1,112(60.0%) |  |  |
| Family annual income per year (RMB) | 100,000 or below | 887(36.4%) | 1,551(63.6%) | 0.018 | 0.894 | 1,048(38.3%) | 1,690(61.7%) | 0.774 | 0.379 | 892(40.6%) | 1,306(59.4%) | 3.518 | 0.061 |
|  | 100,000 or above | 710(36.2%) | 1,252(63.8%) |  |  | 345(36.7%) | 596(63.3%) |  |  | 198(36.2%) | 349(63.8%) |  |  |
| Self-reported health | Poor | 329(44.4%) | 412(55.6%) | 25.31 | < .001 | 312(51.1%) | 299(48.9%) | 54.264 | < .001 | 262(50.9%) | 253(49.1%) | 33.01 | < .001 |
|  | Good | 1,268(34.7%) | 2,391(65.3%) |  |  | 1081(35.2%) | 1987(64.8%) |  |  | 828(37.1%) | 1,402(62.9%) |  |  |
| Chronic diseases | Yes | 120(43.8%) | 154(56.2%) | 7.109 | < .008 | 105(46.9%) | 119(53.1%) | 8.233 | 0.004 | 53(46.9%) | 60(53.1%) | 2.548 | 0.11 |
|  | No | 1,477(35.8%) | 2,649(64.2%) |  |  | 1,288(37.3%) | 2,167(62.7%) |  |  | 1,037(39.4%) | 1,595(60.6%) |  |  |
| Confirmed COVID-19 | Yes | 22(43.1%) | 29(56.9%) | 1.045 | 0.307 | 17(42.5%) | 23(57.5%) | 0.369 | 0.543 | 3(11.5%) | 23(88.5%) | 8.7 | 0.003 |
|  | No | 1,575(36.2%) | 2,774(63.8%) |  |  | 1,376(37.8%) | 2,263(62.2%) |  |  | 1,087(40.0%) | 1,632(60.0%) |  |  |
| Relatives or friends confirmed | Yes | 28(52.8%) | 25(47.2%) | 6.343 | 0.012 | 13(38.2%) | 21(61.8%) | 0.002 | 0.964 | 7(28.0%) | 18(72.0%) | 1.445 | 0.229 |
|  | No | 1,569(36.1%) | 2,778(63.9%) |  |  | 1,380(37.9%) | 2,265(62.1%) |  |  | 1,083(39.8%) | 1,637(60.2%) |  |  |
| Quarantine | Yes | 194(46.1%) | 227(53.9%) | 19.279 | < .001 | 192(47.5%) | 212(52.5%) | 18.005 | < .001 | 130(40.5%) | 191(59.5%) | 0.095 | 0.758 |
|  | No | 1,403(35.3%) | 2,576(64.7%) |  |  | 1,201(36.7%) | 2,074(63.3%) |  |  | 960(39.6%) | 1,464(60.4%) |  |  |
| Perception of COVID-19 | Uncontrolled | 210(41.2%) | 300(58.8%) | 5.944 | < .015 | 192(46.8%) | 218(53.2%) | 15.765 | < .001 | 136(46.4%) | 157(53.6%) |  |  |
|  | Controlled | 1,387(35.7%) | 2,503(64.3%) |  |  | 1,201(36.7%) | 2,068(63.3%) |  |  | 954(38.9%) | 1,498(61.1%) | 6.165 | 0.013 |

Notes: GAD-7, Generalized Anxiety Disorder-Scale-7; COVID-19: Coronavirus Disease 2019; p < .05, statistically significant.
