# Supplementary material for: Prevalence and risk factors of COVID-19-related generalized anxiety disorder among the general public in China: a cross-sectional study
Source: PeerJ. 2023 Jan 18;11:e14720. doi: 10.7717/peerj.14720 (PMC9864122; doi:10.7717/peerj.14720)
Supplement: Supplemental Information 6 — Notes: CI: confidence interval; *semi-partial correlations; **p values were calculated from linear regression models. [file peerj-11-14720-s006.docx]

**Supplemental Table 3 Linear regression analysis of variables related to anxiety in eastern, central and western China**

| **Variable** | ***B*** | ***SE*** | ***Beta*** | ***t*** | ***95%CI*** | **r*** | ***p***** |
| --- | --- | --- | --- | --- | --- | --- | --- |
| **Eastern China** | | | | | | | |
| Age | -0.593 | 0.128 | -0.071 | -4.618 | (-0.844,-0.341) | -0.069 | < .001 |
| Family register | -0.368 | 0.151 | -0.037 | -2.444 | (-0.664,0.073) | -0.036 | 0.015 |
| Self-reported health | 0.915 | 0.157 | 0.089 | 5.82 | (0.607,1.223) | 0.087 | < .001 |
| Chronic diseases | 0.764 | 0.247 | 0.048 | 3.098 | (0.281,1.248) | 0.046 | 0.002 |
| Relatives or friends confirmed | 0.525 | 0.539 | 0.015 | 0.974 | (-.531,1.580) | 0.014 | 0.330 |
| Quarantine | 0.737 | 0.199 | 0.056 | 3.698 | (0.346,1.127) | 0.055 | < .001 |
| Perception of COVID-19 | -0.894 | 0.18 | -0.074 | -4.959 | (-1.248,-.541) | -0.074 | < .001 |
| **Central China** | | | | | | | |
| Gender | -0.517 | 0.13 | -0.065 | -3.988 | (-0.771,-0.263) | -0.064 | < .001 |
| Age | -0.668 | 0.145 | -0.077 | -4.604 | (-0.952,-0.384) | -0.074 | < .001 |
| Self-reported health | 1.288 | 0.178 | 0.121 | 7.222 | (0.938,1.637) | 0.117 | < .001 |
| Chronic diseases | 0.722 | 0.281 | 0.044 | 2.567 | (0.171,1.274) | 0.041 | 0.01 |
| Quarantine | 1.205 | 0.206 | 0.095 | 5.856 | (0.801,1.608) | 0.095 | < .001 |
| Perception of COVID-19 | -1.046 | 0.204 | -0.083 | -5.121 | (-1.447,-0.646) | -0.083 | < .001 |
| **Western China** | | | | | | | |
| Gender | -0.56 | 0.158 | -0.067 | -3.551 | (-0.869,-0.251) | -0.067 | < .001 |
| Age | -0.394 | 0.181 | -0.042 | -2.177 | (-0.749,-0.039) | -0.041 | 0.030 |
| Family register | -0.324 | 0.173 | -0.036 | -1.873 | (-0.663,0.015) | -0.035 | 0.061 |
| Self-reported health | 1.314 | 0.201 | 0.124 | 6.543 | (0.920,1.707) | 0.123 | < .001 |
| Confirmed COVID-19 | -2.022 | 0.805 | -0.047 | -2.512 | (-3.600,-0.443) | -0.047 | 0.012 |
| Perception of COVID-19 | -1.061 | 0.253 | -0.079 | -4.197 | (-1.557,-0.565) | -0.079 | < .001 |
| Notes: CI: confidence interval; *semi-partial correlations; **p values were calculated from linear regression models. | | | | | | | |
