# Supplementary material for: Prevalence and risk factors of COVID-19-related generalized anxiety disorder among the general public in China: a cross-sectional study
Source: PeerJ. 2023 Jan 18;11:e14720. doi: 10.7717/peerj.14720 (PMC9864122; doi:10.7717/peerj.14720)
Supplement: Supplemental Information 7 — Notes: OR: odds ratio; CI: confidence interval; p < 0.05, statistically significant. [file peerj-11-14720-s007.docx]

**Supplemental Table 4 Logistic regression analysis of variables related to anxiety**

| **Variable** | ***B*** | ***SE*** | ***Wald*** | ***OR*** | ***95%CI*** | ***p* value** |
| --- | --- | --- | --- | --- | --- | --- |
| Gender (female/ref: male) | -0.182 | 0.041 | 20.02 | 1.200 | (1.108,1.300) | <.001 |
| Age 40 (39 or below/ref:40 or above) | -0.318 | 0.049 | 42.062 | 1.374 | (1.248,1.513) | <.001 |
| Marital status (unmarried/ ref: married) | 0.019 | 0.049 | 0.153 | 1.020 | (0.925,1.123) | 0.696 |
| Family register (rural/ref: urban) | -0.169 | 0.048 | 12.471 | 1.184 | (1.078,1.301) | <.001 |
| Family annual income (100,000 or above/ref: 100,000 or below) | -0.025 | 0.044 | 0.330 | 0.975 | (0.894,1.063) | 0.566 |
| Self-reported health (poor/ref: good) | 0.514 | 0.053 | 93.686 | 1.672 | (1.507,1.856) | <.001 |
| Chronic diseases (yes/ref: no) | 0.329 | 0.089 | 13.715 | 1.389 | (1.167,1.653) | <.001 |
| Quarantine (yes/ref: no) | 0.311 | 0.064 | 23.615 | 1.365 | (1.204,1.547) | <.001 |
| Perception of COVID-19 (controlled/ref: uncontrolled) | -0.257 | 0.062 | 16.888 | 0.774 | (0.684,0.874) | <.001 |

Notes: *OR*: odds ratio; *CI*: confidence interval; *p* < .05, statistically significant.
