# Supplementary material for: Prevalence and risk factors of COVID-19-related generalized anxiety disorder among the general public in China: a cross-sectional study
Source: PeerJ. 2023 Jan 18;11:e14720. doi: 10.7717/peerj.14720 (PMC9864122; doi:10.7717/peerj.14720)
Supplement: Supplemental Information 8 — Notes: odds ratio; CI: confidence interval; a: The participants have normal level of anxiety (GAD-7 < 5); b: The participants have mild level of anxiety (5 ≦ GAD-7 < 10); c: The participants have moderate and severe level of anxiety (10 ≦ GAD-7 < 21); p < 0.050, statistically significant. [file peerj-11-14720-s008.docx]

**Supplemental Table 5 Ordinal logistic regression** **analysis of variables related to anxiety symptoms**

| **Variable** | **A^a^(n=6744)** | **B^b^(n=3355)** | **C^c^(n=725)** | **Ordinal logistic regression** | | |
| --- | --- | --- | --- | --- | --- | --- |
|  | ***N*(%)** | ***N*(%)** | ***N*(%)** | **OR** | ***95%CI*** | ***p* value** |
| Self-reported health | | | | | | |
| Good | 5,780(85.7%) | 2,652(79.0%) | 525(72.4%) | － | 1.000 | － |
| Poor | 964(14.3%) | 703(21.0%) | 200(27.6%) | 1.674 | (0.415,0.615) | <0.001 |
| Chronic diseases | | | | | | |
| No | 6,411(95.1%) | 3,135(93.4%) | 667(92.0%) | － | 1.000 | － |
| Yes | 333(4.9%) | 220(6.6%) | 58(8.0%) | 1.197 | (0.016,0.344) | 0.031 |
| Quarantine | | | | | | |
| No | 6,114(90.7%) | 2,961(88.3%) | 603(83.2%) | － | 1.000 | － |
| Yes | 630(9.3%) | 394(11.7%) | 122(16.8%) | 1.433 | (0.240,0.481) | <0.001 |
| Perception of COVID-19 | | | | | | |
| Uncontrolled | 675(10.0%) | 363(10.8%) | 175(24.1%) | － | 1.000 | － |
| Controlled | 6,069(90.0%) | 2,992(89.2%) | 550(75.9%) | 0.672 | (-0.515,-0.281) | <0.001 |

Notes: odds ratio; *CI*: confidence interval; a: The participants have normal level of anxiety (GAD-7 < 5); b: The participants have mild level of anxiety (5 ≦ GAD-7 < 10); c: The participants have moderate and severe level of anxiety (10 ≦ GAD-7 < 21); *p* < 0.050, statistically significant.
